# Social Support and Subjective Well-Being in Chinese Parents of Children With Autism Spectrum Disorder: The Mediating Role of Perceived Discrimination

**DOI:** 10.3389/fpsyg.2021.781794

**Published:** 2021-11-08

**Authors:** Yongfei Ban, Ji Sun, Jiang Liu

**Affiliations:** ^1^School of Educational Sciences, Anshun University, Anshun, China; ^2^Faculty of Education, Yunnan Normal University, Kunming, China

**Keywords:** social support, perceived discrimination, subjective well-being, parents with ASD children, mediation analysis

## Abstract

The present research was done to examine whether social support was related to subjective well-being on Chinese parents of children with autism spectrum disorder (ASD) and how perceived discrimination affected this relationship. Two hundred four parents with ASD children were investigated by Inventory of Social Support Behavior, Perceived Discrimination Scale for Parents of Children With ASD, Subjective Well-being Scale. The results showed that perceived discrimination was negatively associated with social support and subjective well-being, and social support was positively related to subjective well-being. Furthermore, perceived discrimination played a partial mediating role between social support and subjective well-being. All the findings suggest that social support can directly influence subjective well-being of parents of ASD children and indirectly influence subjective well-being through perceived discrimination.

## Introduction

Autism spectrum disorder (ASD) is a kind of developmental disability which has been characterized by persistent impairments in social interaction and the existence of repetitive behaviors, interests, and activities (American Psychiatric Association, 2013). Globally, the number of ASD children increases rapidly, and the core symptoms of ASD often persist in life-long development (Centers for Disease Control and Prevention, 2020). Previous studies have suggested that parents raising a child with ASD may experience enormous challenges associated with intensive caregiving tasks and the accompanied comorbidities (e.g., anxiety and depression). In addition, parents may be constantly exposed in public hospitals and centers and were strongly subjected to stigma. Under the high levels of emotional and financial presses, parents with ASD children had low levels of subjective well-being (SWB; Lu et al., 2015; Costa et al., 2017; Garrido et al., 2021). To improving SWB of ASD families, it was essential to explore the potential predictors of SWB for parents with ASD children.

Social support (SS) is presented by the perceived comfort, caring, assistance, and esteem one individual receives from others (Wallston et al., 1983). It may be one of the most beneficial and robust predictors of well-being (Shorey and Lakey, 2011; Hassanein et al., 2021). Previous studies have found that SS is significantly related to families’ well-being (Lin et al., 2011; Frantz et al., 2018; Khusaifan and Keshky, 2020). Furthermore, the parents who had high SS needs reported low levels of well-being (Hassanein et al., 2021).

In addition, members of developmental disabilities families may face discrimination in some social interaction contexts (Schmitt et al., 2014). Perceived discrimination (PD) is understood as the subjective interpretation of unfair and inequitable treatment based on societal group membership (Schmitt et al., 2014). Several studies have shown that PD negatively affects caregiver’s well-being for developmental disabilities families (Zhou et al., 2018; Mitter et al., 2019). For parents with ASD children, PD may lead them to accept stigma as a part of their self-concept (Herek, 2007) and feel blame, anxiety, hopelessness (Recio et al., 2021). The phenomenological variant of ecological systems Theory (PVEST; Spencer et al., 1997) may serve as a theoretical perspective to understand the relationships among SS, PD, and SWB. PVEST emphasized the critical role of coping and adaptive processes in resource access and suggested that coping and adaptive processes affected the individuals’ and families’ appropriate behavior and mental health (Spencer et al., 2015). According to PVEST (Spencer et al., 1997), minority families and individuals might experience stress engagement variables (e.g., perceived social support) when linked with intergroup contact contexts. Furthermore, positive cognitive appraisals of SS would lead to adaptive coping responses (e.g., less discrimination perception, self-acceptance) which influenced behavioral and health-relevant outcomes (e.g., mental health and intimacy). Based on the PVEST, it seems reasonable to assume that PD may mediate the relationship between SS and SWB in parents with ASD children.

However, to our knowledge, previous studies have mainly examined the relationships among SS, PD, and SWB of patients with mental disorders (Jasinskaja-Lahti et al., 2006; Schauman et al., 2019), people with physical disabilities (Ji et al., 2019), and immigrants (Fernández et al., 2014; Hashemi et al., 2019, 2020). Few studies have focused on members of ASD families, especially parents. Thus, it is crucial to understand whether SS is associated with SWB and how PD influence this relationship in the Chinese sample of parents with ASD children.

In summary, this study aims to examine (a) the relationship between SS and SWB and (b) the mediating role of PD in Chinese parents with ASD children. We used measures to assess the levels of SS, PD, and SWB for parents with ASD children. Then, we conducted mediating analysis to examine the mediating role of PD in the relationship between SS and SWB.

## Materials and Methods

### Participants

Two hundred and four parents of children with ASD participated in the present study. The parents were recruited through seven special education centers and schools in the cities of Guiyang, Zunyi, and Liupanshui in China. All of the children had been diagnosed with ASD and received disability-related services in special education centers and schools. The study was approved by the ethics committee of Anshun university. The age of parents ranged from 23 to 48 years (*M* = 34.23, *SD* = 5.78). Most of the parents were female (69.7%) and did not have a university degree (83.3%). The children were between 4–18 years old (*M*_*age*_ = 7.25, *SD*_*age*_ = 3.53). Demographic characteristics can be found in Table 1. The data of six parents were excluded due to the missing data on demographic information and experimental measures. All participants gave written informed consent before the experiment and received a small gift (e.g., a coloring book) to thank them. After the experimental session was finished, the participants were told about the purpose of the study.

**TABLE 1:** Demographic characteristics of participants.

Variable	Mean	SD	Range
Parent’s age	34.23	5.78	23–48
Child’s age	7.25	3.53	4–18

**Variable**			**% (*n*)**

**Parent’s gender**			
Male			30.3(60)
Female			69.7(138)
**Parent’s educational level**			
Primary school and below			11.1(22)
Middle school			27.3(54)
High school degree			23.2(46)
College degree			21.7(43)
University degree and above			16.7(33)

### Measures

#### The Perceived Discrimination Scale for Parents of Children With ASD (PDS-FP)

The PDS-FP was used to measure PD of Chinese parents with ASD children (Zhao, 2018). The scale includes 10 items and uses 4-point scale from 1 (strongly disagree) to 4 (strongly agree). The scale consists of two subscales: discrimination perception and discrimination attribution. Sample items are as follows: “It is hard to make friends”, “Being blamed for not teaching your children well”. The lower scores reflect lower levels of PD. The Cronbach’s α of the scale was 0.91. Internal consistency coefficients of the scale were α = 0.88 in the present study. The previous studies provided good evidence of construct validity for PDS-FP (Zhao, 2018).

#### Inventory of Social Support Behavior

The 10-item Chinese version of the ISSB was used to assess SS of Chinese parents of children with ASD in this study (Barrera et al., 1981; Luo, 2001). Sample items are as follows: “Talked with you about some interests of yours”, “Listened to you talk about your private feelings”. Items are scored on a 3-point scale from 1 (not satisfied) to 3 (satisfied). The lower score of all items indicated that parents reported lower levels of SS. Internal consistency coefficients of the scale reached α = 0.91. In the current study, the Chinese version of ISSB resulted in an internal consistency coefficient of α = 0.88. The previous studies confirmed the construct validity of ISSB (Luo, 2001).

#### The Brief Subjective Well-Being Scale for Chinese Citizen

The brief subjective well-being scale for Chinese citizen [SWBS-CC (brief)] is a 20-items scale used to measure SWB of Chinese parents with ASD children (Xing, 2003). Sample items are as follows: “Compared with the people around me, I feel satisfied.”, “I often get annoyed by some trivial things.”. The items are rated on a 6-point scale from 1 (totally disagree) to 6 (totally agree). The total score can be calculated by averaging all 20 items. The lower score represents the lower levels of SWB. In the present study, parents scoring above 4.5 were considered to have high levels of SWB, and below 2.5 were considered to have low levels of SWB. The Cronbach’s alpha reliability was α = 0.85. In this study, the computed Cronbach’s α was 0.81.

### Procedures

With assist from several special education centers and schools, the research team contacted parents with ASD children who showed interest in participating in this study. Parents were informed the study’s objectives and their right (e.g., protect the anonymity of them). All participants gave clear written informed consent before data collection. Then, an individual meeting was held with each parent to ensure that all parents accurately understood the measures and procedures.

### Data Analysis

The statistical analyses of data were conducted using SPSS software (Version 27.0). At first, Pearson correlation analyses were conducted to examine the relationships between PD, SS, and SWB in parents with ASD children. Then, we used PROCESS macro for SPSS which developed by Hayes to examine the mediation role of PD in the relationship between SS and SWB. The PROCESS macro uses a path analysis modeling tool which is based on regression to assess direct and indirect effects of variables (Hayes, 2018). Following the recommendations of Hayes, we performed the mediation analyses using Model 4 with 5000 bias-corrected bootstrap samples.

## Results

### Descriptive and Correlational Analyses

The mean total scores and correlations of three scales are presented in Table 2. The results showed that the PD was negatively correlated with the SS (*r* = −0.24, *p* < 0.001) and the SWB (*r* = −0.66, *p* < 0.001), indicating that the parents with ASD children reported high levels of PD if they had low levels of SS and SWB. However, the SS positively correlated with SWB (*r* = −0.36, *p* < 0.001). It meant that high levels of SS in parents were associated with high levels of SWB.

**TABLE 2:** Descriptive and correlational analyses (*n* = 198).

	Mean	SD	1	2	3
1 PD	2.24	0.66	—		
2 SS	2.29	0.45	−0.24***	—	
3 SWB	4.01	0.62	−0.66***	−0.36***	—

***p < 0.001.

### The Mediation Role of Perceived Discrimination in the Relationship Between Social Support and Subjective Well-Being

**FIGURE 1:**
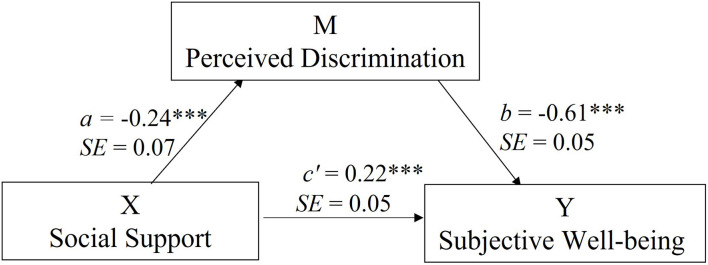
The mediation model for the effect of PD in the relationship between SS and SWB. ***p < 0.001.

To test whether PD would mediate the relationship between SS and SWB, we ran a mediation analysis with SS as a predictor, PD as a mediator and SWB as the dependent variable. Figure 1 presents the mediation model. The results showed that the effect of SS on PD was −0.24 (SE = 0.07, *t* = −3.43, *p* < 0.001, 95% CI: −0.38 to −0.10). Also, the effect of PD on SWB was −0.61 (SE = 0.05, *t* = −11.43, *p* < 0.001, 95% CI: −0.71 to −0.50). Importantly, SS has a total effect on SWB of 0.36 (SE = 0.07, *t* = 5.46, *p* < 0.001, 95% CI: 0.23 to 0.50). Specifically, the indirect effect of SS on SWB was 0.15 and statistically different from zero (95% CI: 0.04 to 0.25), while the direct effect was 0.22 (SE = 0.05, *t* = 4.11, *p* < 0.001, 95% CI: 0.11 to 0.32). This model explained 48.01% of SWB variance (*F* = 90.02, *p* < 0.001). Overall, these results suggested that PD partially mediated the relationship between SS and SWB.

## Discussion

The current study aimed to examine the relationship between SS and SWB and the mediating role of PD in Chinese parents of children with ASD. First, the findings showed that SS was positively related to SWB. Specifically, parents with ASD children who perceived high levels of SS reported high levels of SWB. These results conform with previous studies (Lin et al., 2011; Frantz et al., 2018; Khusaifan and Keshky, 2020). From the theoretical point of view, the main effects of SS suggested that the presence of SS may provide important contributions to positive mental health and SWB (Kondrat et al., 2018). Parents with ASD children value SS as an active coping strategy against conflicting life events (Davis and Brekke, 2014). As positive social-psychological resources of families and individuals, SS leads parents to accept children’s condition and positively deal with the complex challenges accompanying the rearing of ASD children (Ludlow et al., 2012). For ASD families, an intense and active system of SS is one of the most influential factors that can improve SWB of families.

Second, the present study’s finding suggested a significant negative relationship between PD and SWB. High levels of PD were associated with low levels of SWB for Chinese parents of children with ASD. This finding is consistent with the previous studies (Ji et al., 2019; Schauman et al., 2019). For ASD families, facing stigmatization and discrimination from their environment intensified the perceived stress and reinforced the feeling of being rejected, self-blame, and angry of individuals (Yasuhiro, 2018). Furthermore, PD might make it difficult for families’ members to seek supportive resources (Benomir et al., 2016). On the other hand, the members of ASD families would employ ineffective coping strategies (e.g., avoiding interpersonal contacts) to deal with PD and further exacerbated the severe negative impact on families’ social networks (Kondrat et al., 2018). In this manner, PD harmed the SWB of ASD families (Schmitt et al., 2014).

Third, and most importantly, the finding of this study indicated the mediating role of PD. More specifically, PD mediated the association between SS and SWB. The results supported the view of the PVEST. As PVEST pointed out, the balance between protective and risk factors in individuals’ and families’ development produced specific psycho-social outcomes. The SS was an important protective factor that buffered against the stress of individuals and families. In addition, the self-evaluation process (e.g., PD), which indicated the perception of one’s association with others in social interactions (e.g., the members of hospitals, neighbors), had a substantial effect on mental health outcomes (Spencer et al., 2015). When contacting with other people, inappropriate supports may lead people from ASD families to experiencing negative stereotypes about them and their families, such as the belief that “the burden of disability is unending for the family and they are the most perfect objects of charity” (Block, 2018). Parents and their ASD children may have to bear all the pressure associated with the unsuitable and excessive assistance from fully human people, especially in collectivist societies (e.g., China and Japan) where helping people with disabilities in public places is an important social norm. As Liao et al. (2019) pointed out, the PD related to social interactions had a negative effect on SWB for parents with ASD children.

Finally, these findings should be interpreted cautiously, and there are some limitations in the present study. First, all the data of this study were based on the parents’ self-reports about PD, SS, and SWB, and our study lacked the quantitative indexes of these variables. Second, although this study provides evidence of the relationships between SS, PD, and SWB, it is also essential to know how these relationships change and what factors influence them. In addition, this study was cross-sectional design for parents with ASD children. Still, it remains unclear whether the relationship between SS and SWB and the mediating role of PD could also be obtained in samples of parents with normal children. Future research may examine these issues.

## Conclusion

To our knowledge, this is one of few studies to examine the relationships among PD, SS, and SWB in Chinese parents of children with ASD. The results supported the relationship between SS and SWB. Furthermore, PD performed as a mediator of this relationship. These findings may provide valuable information on how to improve well-being in Chinese parents of children with ASD. Given the mediating role of PD, programs related to PD should be designed to enhance well-being in Chinese parents of children with ASD who report high levels of SS. In addition, consider the direct effect of SS on SWB, interventions aiming at improving SS should be implemented to increase well-being in Chinese parents of children with ASD.

## Data Availability Statement

The data presented in this study are available on request from the corresponding author. The data are not publicly available due to the privacy of the participants.

## Ethics Statement

The studies involving human participants were reviewed and approved by School of Education Science, Anshun University. The patients/participants provided their written informed consent to participate in this study.

## Author Contributions

JL, JS, and YB performed the research, collected data, analysis, and interpretation of data. JS and YB wrote the manuscript and designed the research. All authors contributed to the article and approved the submitted version.

## Conflict of Interest

The authors declare that the research was conducted in the absence of any commercial or financial relationships that could be construed as a potential conflict of interest.

## Publisher’s Note

All claims expressed in this article are solely those of the authors and do not necessarily represent those of their affiliated organizations, or those of the publisher, the editors and the reviewers. Any product that may be evaluated in this article, or claim that may be made by its manufacturer, is not guaranteed or endorsed by the publisher.
